# ST-Elevation Myocardial Infarction in a 27-Year-Old Male With COVID-19

**DOI:** 10.7759/cureus.10384

**Published:** 2020-09-11

**Authors:** Mohammed Ali, Aisha Mujahid, Khalid Sherani, Salim Surani

**Affiliations:** 1 Pulmonary/Critical Care Medicine, Corpus Christi Medical Center, Corpus Christi, USA; 2 Medicine, Shadan Institute of Medical Sciences, NTR University of Health Sciences, Hyderabad, IND; 3 Internal Medicine, Corpus Christi Medical Center, Corpus Christi, USA; 4 Internal Medicine, University of North Texas, Dallas, USA

**Keywords:** sars-cov-2, covid-19, myocardial injury, st-elevation myocardial infarction (stemi)

## Abstract

Severe acute respiratory syndrome coronavirus 2 (SARS-CoV-2) is a virus that led to a global public health emergency causing coronavirus disease 2019 (COVID-19). It was initially identified in Wuhan, China after causing significant respiratory illness. Although respiratory symptoms are the most common presenting symptoms, it is now recognized that COVID-19 encompasses multiple organ systems including the cardiovascular system. Acute myocardial injury and ST-elevation myocardial infarction (STEMI) have now been associated with COVID-19. COVID-19 patients with cardiovascular manifestations are at risk for increased severity of illness. Here we present a case of a very young 27-year-old patient without any past history of hypertension, coronary artery disease, or any risk factors for coronary artery disease except obesity, who developed STEMI while in the hospital.

## Introduction

Coronavirus is an enveloped positive sense RNA virus belonging to the family Coronaviridae. Coronaviruses are widespread among birds and mammals. However, two subtypes (genera) of the subfamily Orthocoronavirinae cause a significant disease in humans: Alphacoronavirus and Betacoronavirus [1]. Severe acute respiratory syndrome coronavirus 2 (SARS-CoV-2) is a novel coronavirus with phylogenetic similarity to SARS-CoV. SARS-CoV-2 belongs to the Betacoronavirus similar to SARS-CoV and Middle East respiratory syndrome coronavirus (MERS-CoV), which are two related viruses that also caused significant epidemics with mortality rates of 10% and 35%, respectively [1,2]. In December 2019 in the city of Wuhan in Hubei province of China, officials identified a cluster of cases of respiratory infection of unknown etiology [3]. This was later identified to be SARS-CoV-2. The virus spread very rapidly through the city and subsequently out of the country to the rest of the world. The disease caused by the virus was subsequently termed coronavirus disease 2019 (COVID-19) by the World Health Organization in February 2020 [4]. It has since been declared a pandemic and has wreaked havoc around the world, leading to an unprecedented public health response on a global scale. The cluster of symptoms caused by the SARS-CoV-2 virus is similar to any viral pneumonia causing dyspnea, fever, cough, malaise, and myalgia. The most common reason for hospitalization by far is respiratory failure and in severe cases acute respiratory distress syndrome (ARDS). Most cases are thought to be very mild not requiring hospitalization; however, of the hospitalized cases, there have been numerous cases of cardiac complications besides just respiratory illness. Here we present a case of ST-elevation myocardial infarction (STEMI) in a very young 27-year-old African American patient who was admitted for respiratory failure secondary to COVID-19.

## Case presentation

A 27-year-old obese African-American male with no significant past medical history presented to the emergency department (ED) with the complaint of dry non-productive cough, shortness of breath, and fever with a maximum temperature of 102.9 degrees Fahrenheit prior to arrival. He reported visiting his sister in a different city who had tested positive for SARS-CoV-2 virus and was diagnosed with COVID-19. The patient stated shortly after he started having symptoms, which included shortness of breath, cough, nausea, vomiting, and diarrhea. These symptoms progressively became worse over the next eight days, and he now presented with significant dyspnea.

**Figure 1:**
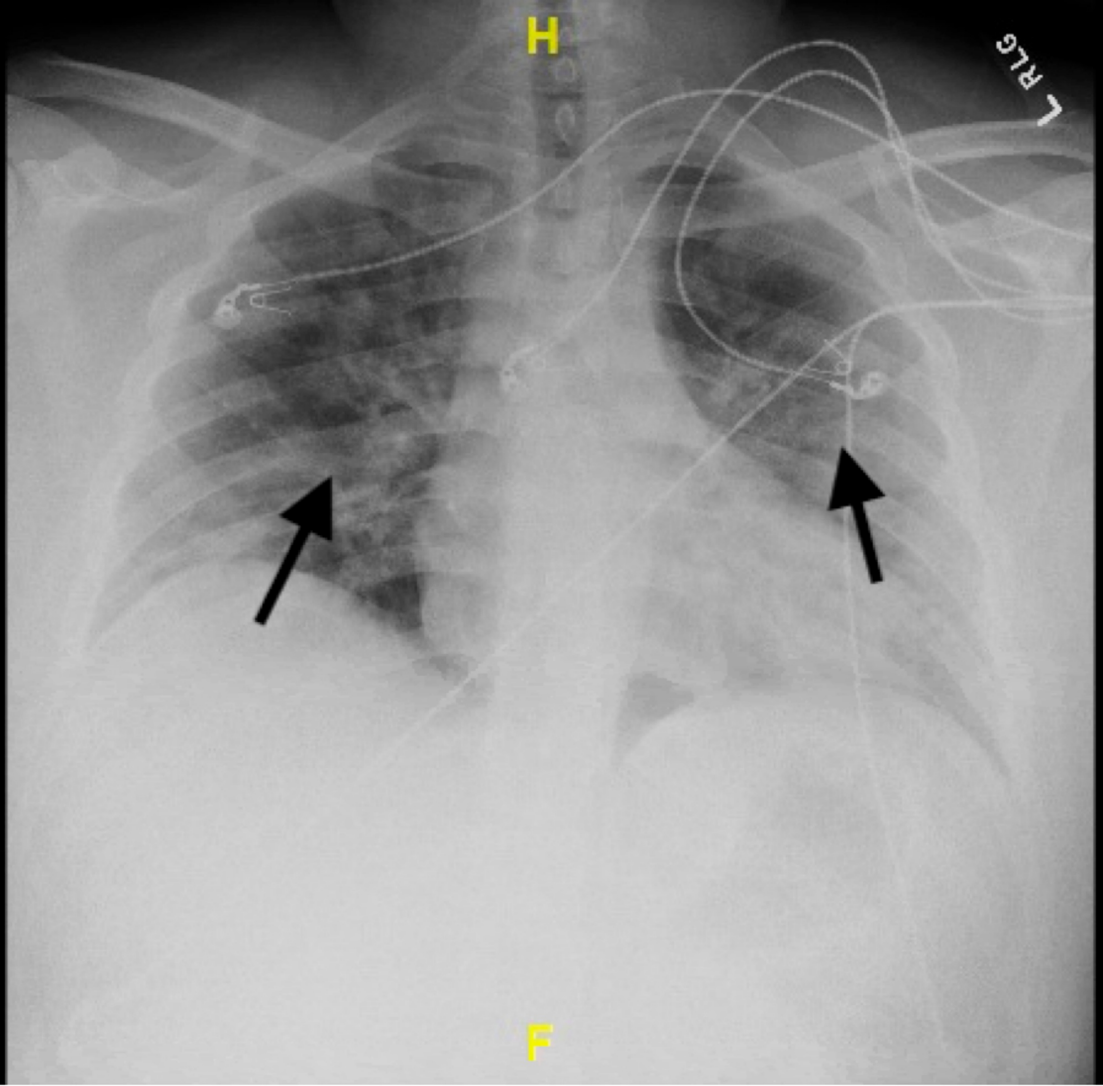
One view chest X-ray on presentation revealing bilateral patchy infiltrates.

**Figure 2:**
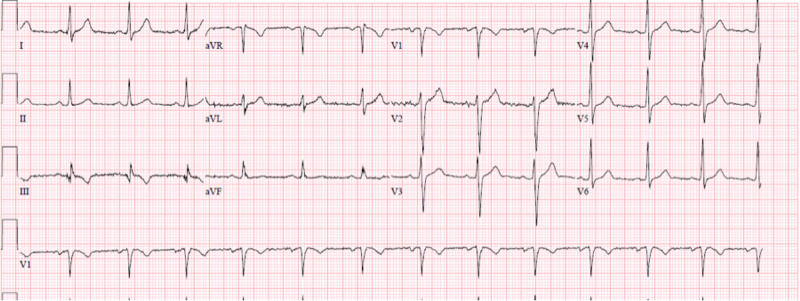
Normal 12-lead electrocardiogram (EKG) on presentation.

Upon presentation in the ED, he was immediately noted to be hypoxic with pulse oxymetry revealing oxygen saturation of 81% on room air. The rest of his vital signs were within normal limits. He was immediately placed on airborne precautions and started on supplemental oxygen via nasal canula receiving five liters per minute (LPM). A one-view chest X-ray was obtained, which revealed bilateral patchy infiltrates (Figure 1). An electrocardiogram (EKG) was also obtained which was normal without ST-segment changes (Figure 2). Initial laboratory workup revealed white blood cell count of 9.5 × 10^3^/µL with 7.2% lymphocytes and an absolute lymphocyte count of 0.68 × 10^3^/µL. D-dimer was 396 ng/mL. Complete metabolic panel (CMP) revealed potassium of 3.2 mmol/L, blood urea nitrogen (BUN) 12 mg/dL, and creatinine 1.0 mg/dL. All were within normal limits besides potassium, which was supplemented in the ED. Nasal swab for nucleic acid amplification of SARS-CoV-2 RNA was positive. He was then admitted to the intensive care unit (ICU) for close monitoring given his hypoxia and potential for deterioration.

**Figure 3:**
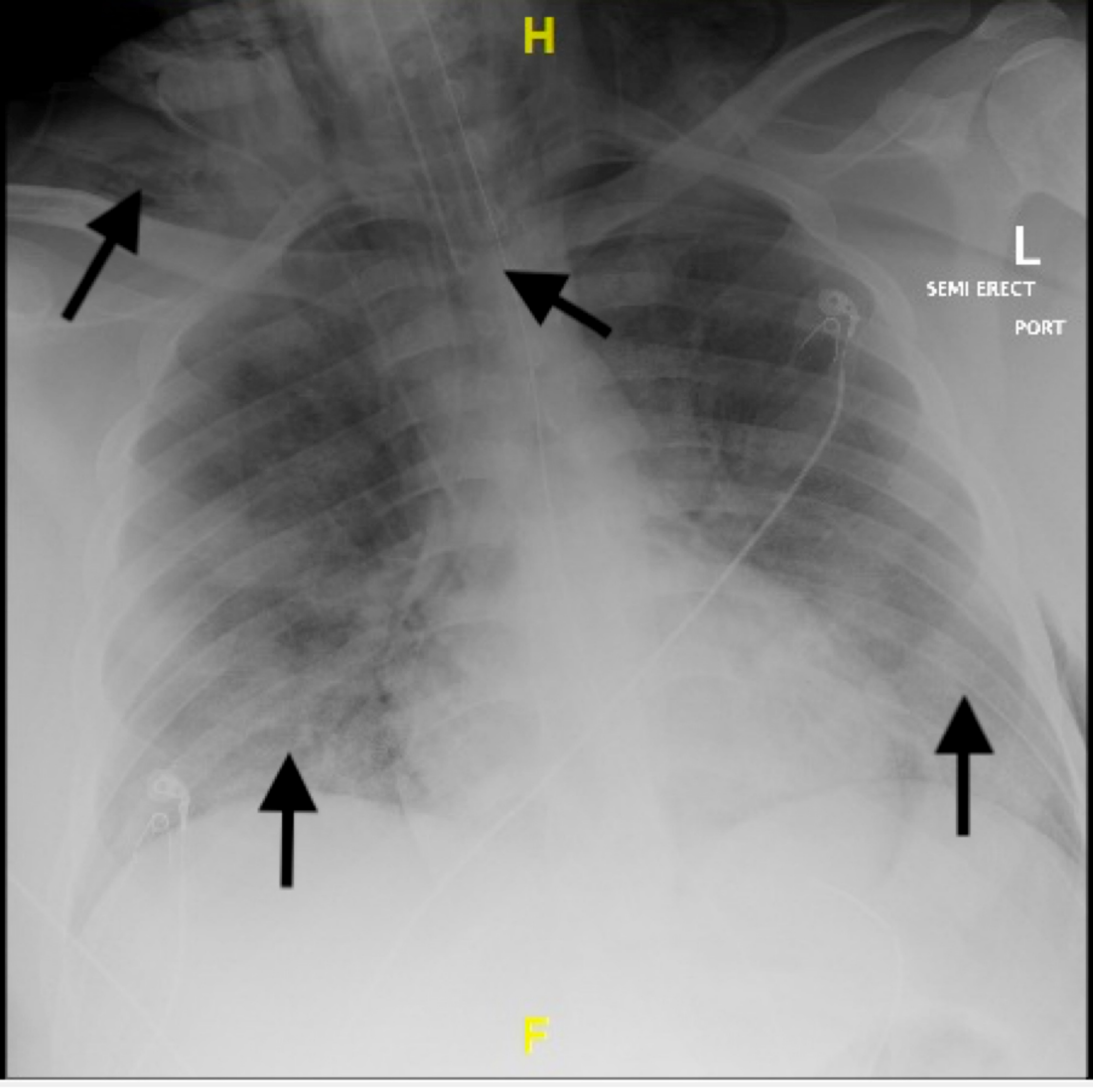
Post-intubation chest X-ray revealing endotracheal tube in appropriate position and slight worsening of the bilateral infiltrates along with subcutaneous emphysema.

He was started on dexamethasone 6 mg intravenous daily, azithromycin 500 mg orally daily, vitamin C 2,000 mg every orally every six hours, zinc sulfate 220 mg orally twice daily, vitamin D 1,000 IU orally daily, and thiamine 100 mg orally twice daily. He was also initiated on weight-based low molecular weight heparin at 1 mg/kg. Remdesivir was unfortunately not available at this time for the patient. During the patient’s first night, his oxygen requirements increased and he required 10 LPM to maintain oxygen saturation around 90%. He was transfused two units of convalescent plasma on hospital day 1. On hospital day 2, he was requiring a non-rebreather mask (NRBM) to maintain the same saturation. An arterial blood gas (ABG) was obtained that revealed pH of 7.43, pCO_2_ 40.5 mmHg, and pO_2_ 71 mmHg on 100% FiO_2_ with NRBM . His respiratory status continued to worsen, and on hospital day 3 he was placed on non-invasive ventilation on which he remained until the following night when his respiratory status further deteriorated requiring intubation and mechanical ventilation. A post-intubation chest X-ray was obtained (Figure 3), which again revealed worsening infiltrates and also some subcutaneous emphysema in the soft tissues of the neck and bilateral apices. 

**Figure 4:**
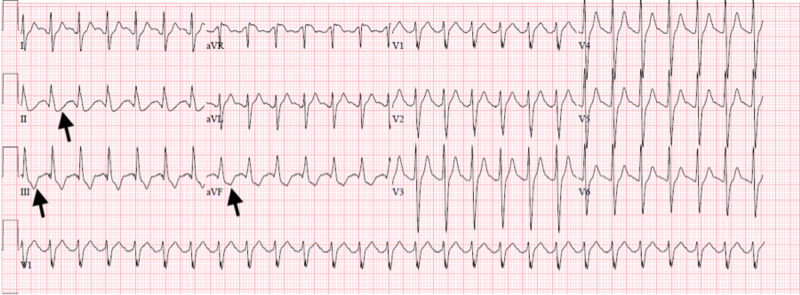
Twelve-lead electrocardiogram (EKG) with arrows pointing to ST-segment depressions in leads II, III, and AVF.

All his labs remained within normal limits except for D-dimer, which increased to 886 ng/mL. He remained stable on the ventilator the following day, and ABG on hospital day 5 revealed pH 7.31, pCO_2_ 39.1 mmHg, and pO_2_ 131.2 mmHg on the following settings: pressure control with respiratory rate of 24, inspiratory pressure of 26 cmH_2_O, and positive end-expiratory pressure (PEEP) of 18 cmH_2_O. The following morning on hospital day 6, it was noted by nursing staff that his rhythm had changed all of a sudden. A Stat EKG (Figure 4) was obtained that revealed ST depression in the inferior leads II, III, and AVF. Stat labs were also obtained, and CMP revealed acute renal failure with BUN 60 mg/dL and creatinine 5.95 mg/dL, both of which were normal the day prior. 

**Figure 5:**
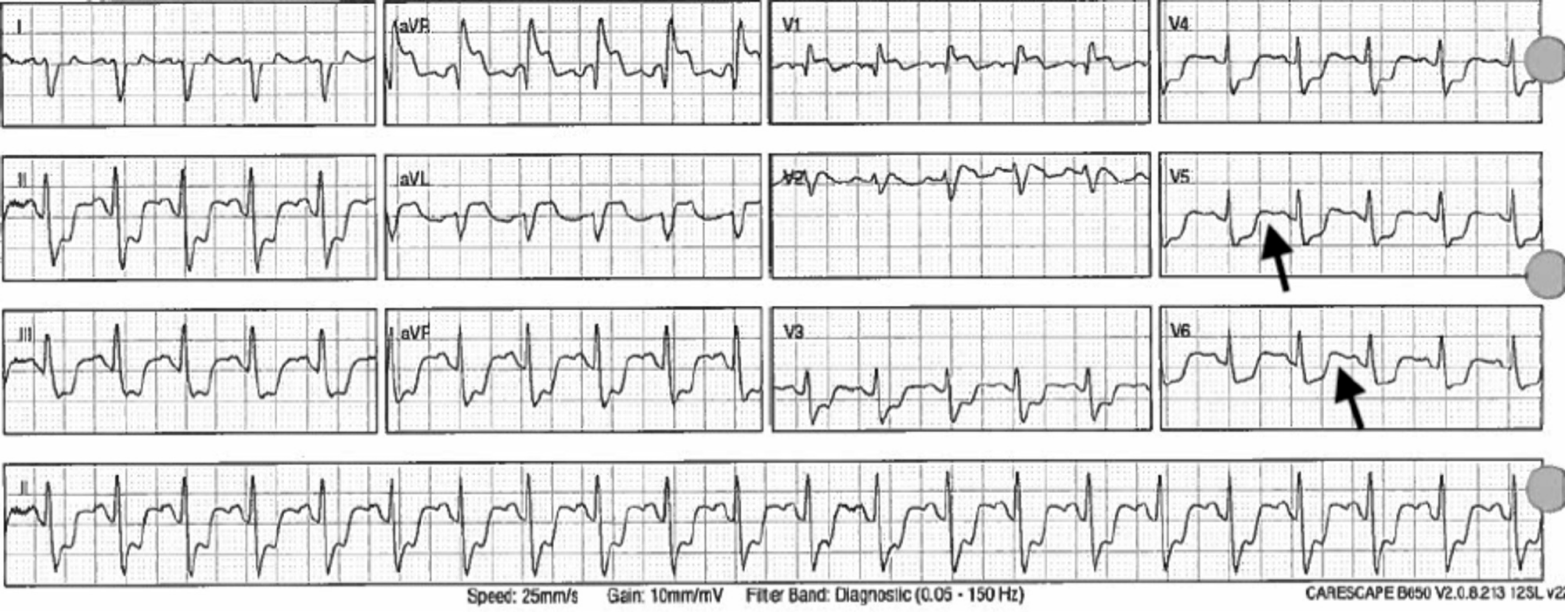
Twelve-lead electrocardiogram (EKG) revealing ST-segment elevation in the lateral leads.

Another rhythm change was observed on the telemetry monitor; therefore, a repeat EKG (Figure 5) was obtained that revealed an acute STEMI in the inferior and lateral leads. Code STEMI was called overhead, the heart catheterization lab was activated, and cardiology consulted. However, within a few minutes of activation, the patient went into cardiac arrest. Advanced cardiac life support was then initiated, but unfortunately after aggressive resuscitation efforts the patient was pronounced dead. 

## Discussion

Here we described a case of a young 27-year-old male with only history of obesity who initially presented with dyspnea due to COVID-19. He eventually suffered an STEMI and unfortunately succumbed to this dreadful disease. It should be reported that, to the best of our knowledge, this was the youngest COVID-19 patient reported to have suffered an STEMI. Since the start of the COVID-19 pandemic, much has been learned about the disease process. However, much remains to be learned. The pathophysiology of the disease remains elusive.

SARS-CoV-2 virus predominantly causes respiratory illness, such as respiratory failure, pneumonia, and ARDS. It is now increasingly clear however that there are cardiovascular complications, such as myocardial injury, arrhythmia, acute heart failure, and venous thromboembolism, involved with COVID-19 that occur more frequently than previously thought [5]. The extrapulmonary and systemic manifestations of COVID-19 remain very poorly understood. In a recent cohort retrospective study consisting of 419 patients, Shi et al. reported that cardiac injury, as defined by serum cardiac enzyme, troponin I, above the 99th percentile upper reference limit, was present in 19.7% of the patients [6]. Moreover, studies have shown that patients with pre-existing cardiovascular disease who are diagnosed with COVID-19 have an increased risk of not only contracting the disease but also having a severe form of it or even death [7,8].

The pathophysiology of the cardiovascular injury in patients with COVID-19 is not well understood. Several mechanisms have been postulated by which SARS-CoV-2 can cause cardiovascular complications. SARS-CoV-2 infection occurs through receptor-mediated endocytosis triggered by binding of the viral spike protein to the angiotensin-converting enzyme 2 (ACE-2) receptor on the host cell. The ACE-2 receptor is highly expressed in myocardial tissue, which may serve as a direct route of invasion for the virus into myocardial tissue causing disease [9]. The virus can also cause systemic inflammation leading to cytokine storm, which can result in multiorgan system failure, including the cardiovascular system [7,10]. Respiratory failure and the resulting hypoxemia can also lead to increasing demand-supply mismatch, thus leading to acute myocardial injury. SARS-CoV-2 is also widely thought to provoke a prothrombotic state leading to microthrombi formation, which can then embolize leading to acute ischemic event of the target end organ [8]. This mechanism is what is thought to have occurred in our young patient.

A recent retrospective analysis by Stefanini et al. revealed that STEMI was the presenting clinical manifestation in 24 out of 28 COVID-19 patients who were diagnosed with an STEMI. The rest had STEMI during the course of the hospitalization. It should be noted that the mean age of these patients was 68 ± 11 years, ranging from 45 to 89 years [11]. In 40% of the cases, a culprit lesion was not identified on coronary angiography. Another case series published by Bangalore et al. identified 18 patients with COVID-19 who had a STEMI. The median age was 63 years, ranging from 54 to 73 years. One-third of the patients did not have obstructive disease [12]. The exact pathophysiological mechanisms of myocardial injury remains unclear; however, as mentioned microthrombi, cytokine storm, and hypoxic injury are thought to be the main means along with plaque rupture, coronary spasm, or direct endothelial or vascular injury. It was however noted that cardiovascular complications in patients with COVID-19 led to a higher morbidity and mortality rate [8,11-13].

## Conclusions

COVID-19 has sparked a worldwide public health emergency and has led to significant morbidity and mortality globally. COVID-19 has now been associated with increased cardiovascular injury and even more so in patients with severe disease. Although the exact pathophysiology of cardiac injury remains a mystery, it is important to promptly recognize this manifestation. As our case illustrates young age, it may not preclude patients from cardiac injury resulting in significant morbidity and even death.
